# Integrative statistical analyses of multiple liquid biopsy analytes in metastatic breast cancer

**DOI:** 10.1186/s13073-021-00902-1

**Published:** 2021-05-17

**Authors:** Corinna Keup, Vinay Suryaprakash, Siegfried Hauch, Markus Storbeck, Peter Hahn, Markus Sprenger-Haussels, Hans-Christian Kolberg, Mitra Tewes, Oliver Hoffmann, Rainer Kimmig, Sabine Kasimir-Bauer

**Affiliations:** 1 Department of Gynecology and Obstetrics, University Hospital of Essen, Hufelandstr. 55, 45122 Essen, Germany; 2 QIAGEN GmbH, 40724 Hilden, Germany; 3 Department of Gynecology and Obstetrics, Marienhospital Bottrop, 46236 Bottrop, Germany; 4 Department of Medical Oncology, University Hospital of Essen, 45122 Essen, Germany

**Keywords:** Multi-parametric, Multi-layer, Multi-modal, Multi-analyte, Liquid biopsy, Metastatic breast cancer patients

## Abstract

**Background:**

Single liquid biopsy analytes (LBAs) have been utilized for therapy selection in metastatic breast cancer (MBC). We performed integrative statistical analyses to examine the clinical relevance of using multiple LBAs: matched circulating tumor cell (CTC) mRNA, CTC genomic DNA (gDNA), extracellular vesicle (EV) mRNA, and cell-free DNA (cfDNA).

**Methods:**

Blood was drawn from 26 hormone receptor-positive, HER2-negative MBC patients. CTC mRNA and EV mRNA were analyzed using a multi-marker qPCR. Plasma from CTC-depleted blood was utilized for cfDNA isolation. gDNA from CTCs was isolated from mRNA-depleted CTC lysates. CTC gDNA and cfDNA were analyzed by targeted sequencing. Hierarchical clustering was performed within each analyte, and its results were combined into a score termed Evaluation of multiple Liquid biopsy analytes In Metastatic breast cancer patients All from one blood sample (ELIMA.score), which calculates the contribution of each analyte to the overall survival prediction. Singular value decomposition (SVD), mutual information calculation, k-means clustering, and graph-theoretic analysis were conducted to elucidate the dependence between individual analytes.

**Results:**

A combination of two/three/four LBAs increased the prevalence of patients with actionable signals. Aggregating the results of hierarchical clustering of individual LBAs into the ELIMA.score resulted in a highly significant correlation with overall survival, thereby bolstering evidence for the additive value of using multiple LBAs. Computation of mutual information indicated that none of the LBAs is independent of the others, but the ability of a single LBA to describe the others is rather limited—only CTC gDNA could partially describe the other three LBAs. SVD revealed that the strongest singular vectors originate from all four LBAs, but a majority originated from CTC gDNA. After k-means clustering of patients based on parameters of all four LBAs, the graph-theoretic analysis revealed CTC *ERBB2* variants only in patients belonging to one particular cluster.

**Conclusions:**

The additional benefits of using all four LBAs were objectively demonstrated in this pilot study, which also indicated a relative dominance of CTC gDNA over the other LBAs. Consequently, a multi-parametric liquid biopsy approach deconvolutes the genomic and transcriptomic complexity and should be considered in clinical practice.

**Supplementary Information:**

The online version contains supplementary material available at 10.1186/s13073-021-00902-1.

## Background

Liquid biopsy provides markers to assess the tumoral heterogeneity across oncological treatments with minimal invasion [1]. In breast cancer (BC), the leading form of cancer in women worldwide [2], diverse liquid biopsy analytes (LBAs) have been proven to harbor relevance in clinical practice. For example:

The number of circulating tumor cells (CTCs) [3, 4] as well as the concentration of cell-free tumor DNA (ctDNA) [5] has been proven to correlate significantly with overall survival (OS).

Molecular characterization has identified stemness signatures at a messenger RNA (mRNA) level in extracellular vesicles (EVs) to be associated with decreased survival time in metastatic BC (MBC) patients [6].

Profiling CTCs revealed HER2 overexpression in the blood of patients with HER2-negative primary tumors, and although clinical effectiveness has not yet been concretely proven, it points towards a possible targeted anti-HER2 therapy based on blood testing [7–9].

A detailed characterization of variants occurring in the ctDNA can be used for therapy monitoring, as shown by the use of *ESR1* variants (found in cell-free DNA (cfDNA) and occurring during aromatase inhibitor therapy) as indicators of disease progression [10, 11].

Additionally, cfDNA variant evaluation can also be used for personalized therapy decisions in hormone receptor-positive (HR+) MBC patients since Alpelisib, a PI3Kα inhibitor, was approved for patients with *PIK3CA* mutant tumors [12].

It is worth noting that most studies have focused on single LBAs. Pairwise comparisons of maximally two orthogonal LBAs in matched samples, especially with regard to their molecular characterization, revealed the additive value of the simultaneous use of more than one LBA in MBC [13–19]. To extend these findings, we speculated whether the insights hidden in numerous independent datasets—when integrated properly—might be greater than their individual sums. Consequently, a multi-parametric dataset might enable maximizing the information available for deliberated therapy management. Therefore, we sought to find an optimal way to leverage and integrate transcriptional and genomic information from multiple liquid biopsy reservoirs.

**Fig. 1:**
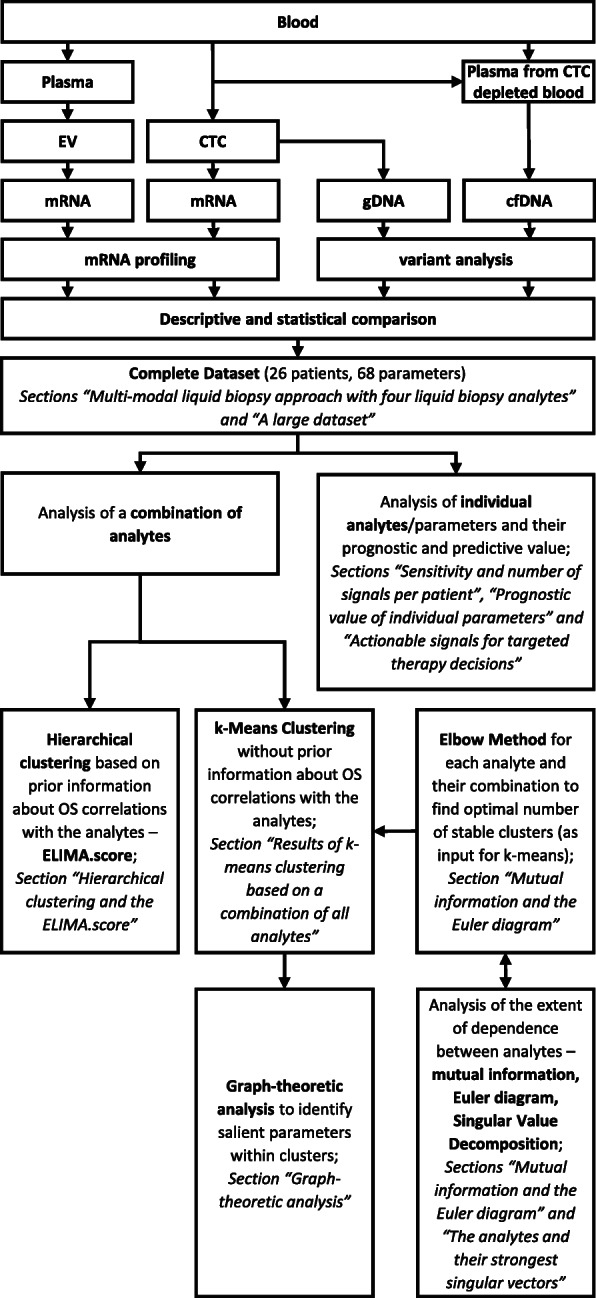
Study design. EV mRNA, CTC mRNA, CTC gDNA, and cfDNA were isolated from only 18ml of blood and mRNA profiling or variant profiling resulted in comparable data sets for comprehensive integration. Integrative statistical analyses performed included the analysis of the individual analytes and their prognostic value, while the combination of the analytes was analyzed from the section “Hierarchical clustering and the ELIMA.score” onwards. While hierarchical clustering was performed with prior knowledge about OS correlations, k-means clustering was performed without prior correlation information. A prerequisite for k-means clustering was determining the optimal number of stable clusters that could be formed, which was found by the elbow method. Mutual information and SVD analyses were carried out to determine whether or not it was necessary to use all the LBAs. With the results of the k-means clustering, we performed a graph-theoretic analysis to identify the salient parameters within the clusters

The resulting project was called *ELIMA* and stands for *E*valuation of multiple *L*iquid biopsy analytes *I*n *M*etastatic breast cancer patients *A*ll from one blood sample. Practically, a workflow had to be implemented that enabled parallel isolation and analysis of CTC mRNA, EV mRNA, CTC genomic DNA (gDNA), and cfDNA from a minimized blood volume to guarantee patient compliance. We then compared all four LBAs from blood drawn at the time of disease progression in a stringent HR+ HER2− MBC cohort (*n*=26) with descriptive and rigorous statistical approaches (Fig. 1). A metric called the ELIMA.score was defined to examine the prognostic value of a multi-modal approach. This was followed by further statistical analyses to examine the interplay between the LBAs.

## Methods

### Patients

Blood samples from 26 MBC patients were studied. All participants were ≥18 years and had Eastern Cooperative Oncology Group (ECOG) scores for performance status of 0–2; no severe, uncontrolled co-morbidities, or medical conditions; and no second malignancies. Prior treatment, radiation, all kinds of surgical intervention, or any other treatment of BC was permitted. MBC patients had estrogen (ER) and/or progesterone (PR) receptor-positive primary tumors [summarized as hormone receptor-positive (HR+)]. Furthermore, all included patients had primary tumors with (a) <10% of HER2 expressing tumor cells (DAKO score 0) or (b) with HER2 expressing cells without complete membrane staining (DAKO score 1) or (c) tumors with DAKO score 2, but without ERBB2 overamplification (by in situ hybridization) (*n*=22). Patients with ER-positive and/or PR-positive and HER2-negative metastases were also included if their ER, PR, and HER2 status of the primary tumor was unknown (*n*=4). All patients showed a progressive MBC at the time of blood draw evaluated by radiologic staging via CT or MRI. Patient characteristics are available in Additional file 1: Table S1.

The study was conducted at the Department of Gynecology and Obstetrics, in collaboration with the Department of Medical Oncology, both at the University Hospital Essen, Germany, with the Marienhospital Bottrop, Germany (for specimen recruitment), and with QIAGEN GmbH, Hilden, Germany (for library preparation and sequencing analysis). In accordance with the Declaration of Helsinki, written informed consent was obtained from all participants at enrollment, and specimens were collected using protocols approved by the Ethics Committee of the University Hospital of Essen (12-5265-BO).

### Sample collection and liquid biopsy analyte extraction

Eighteen milliliters of EDTA blood was collected and CTCs were isolated in duplicate from 5 ml of whole blood by positive immunomagnetic selection (AdnaTest EMT-2/StemCell Select^TM^, QIAGEN) [13]. CTC-depleted blood remaining after positive immunomagnetic selection [20] as well as the remaining blood (not used for CTC isolation) were centrifuged and stored. EVs were isolated from pre-filtered plasma by affinity-based binding to a spin column [13, 21]. Subsequently, the total RNA was isolated and purified (exoRNeasy Kit, QIAGEN). The mRNA was isolated from the CTC lysates and from the vesicular RNA eluates by Oligo(dT)_25_ beads [13]. The supernatant remaining from the CTC lysates after incubation with the Oligo(dT)_25_ beads, called the mRNA-depleted CTC lysate, was used to isolate the gDNA using the AllPrep DNA/RNA Nano Kit prototype (QIAGEN) [14]. cfDNA was isolated by affinity-based binding to magnetic beads (QIAamp MinElute ccfDNA Kit, QIAGEN) using plasma from CTC-depleted blood [20]. Buffy coat DNA and normal tissue DNA that was available (from 18 of the 26 patients) was used as matched germline control. Detailed protocols are available in Additional file 2.

### Quantitative PCR

Purified mRNA was reverse transcribed. The AdnaTest TNBC Panel prototype (QIAGEN), consisting of multi-marker real-time quantitative PCR (RT-qPCR) assays, has been described in detail [13]. Pre-amplified cDNA was analyzed for one of the 17 transcripts with the StepOnePlus™ (Life Technologies) real-time system. Potential PCR inhibition and contamination were checked, and data evaluation was performed with normalization to *PTPRC* and data from healthy donor controls [13] (Additional file 2).

### Sequencing

The libraries were constructed with a customized QIAseq Targeted DNA Panel Kit (QIAGEN) targeting all exonic regions of 17 genes. Library preparation using cfDNA or CTC gDNA was previously described in detail [14]. In brief, the preferred cfDNA input of 30–60 ng and the entire CTC gDNA eluate (20 μl) was used for library preparation with no prior quantification. Library preparation included end-repair, a-addition, and enzymatic fragmentation (only for CTCgDNA). The volumes of barcoded adapters, including unique molecular indices (UMIs) and sample-specific indices, used for ligation to CTCgDNA are five times the volumes required for ligation to cfDNA. Targeted enrichment and universal PCR amplification were performed. All pooled libraries were analyzed by paired-end sequencing on an Illumina NextSeq instrument.

Bioinformatical analysis of the raw sequencing data of cfDNA and CTC gDNA was performed on the basis of the pipeline previously described [14]. Exclusion criteria, such as the minimal number of read fragments, a minimal UMI coverage, and a uniform UMI coverage of the target regions, were defined and are listed in Additional file 2. The input amount, library yield, and sequencing quality parameters for each sample are summarized in Additional file 3: Table S2. For the analysis, we used QIAGEN’s GeneGlobe and Ingenuity Variant Analysis (IVA; QIAGEN) for annotation, scoring, filtering, and interpretation of the resulting variant files. All filter settings were described in detail [14] including the confidence filter that excludes flagged variants from smCounter2 software, the common variant filter that excludes variants with a prevalence of >3% in the normal population, and the cancer driver variant filter that excludes, among others, variants with a prevalence of >0.01% in the TCGA or COSMIC databases.

Original raw sequencing data are available at the European Nucleotide Archive with the study accession number PRJEB39331 [22], and all called variants and their corresponding allele frequencies are listed per patient and per analyte in Additional file 4: Table S3.

Germline control samples were prepared with the same library protocol as described for CTC gDNA, but the sequencing depth for the germline controls was lower when compared to cfDNA and CTC gDNA sequencing. In Additional file 4: Table S3, cfDNA and CTCgDNA variants detected in the germline control have been depicted using different colors.

### The ELIMA.score and further statistical analyses

For improved readability, the order in which the integrative statistical analyses used in this study were carried out and their relationship to one another are depicted in a flowchart (Fig. 1). The Kaplan–Meier estimator (log-rank test) and Cox regression models were used to assess OS.

Hierarchical clustering according to Ward’s method with Euclidean distance was conducted as follows. Step 1: First, all LBAs were analyzed separately by means of hierarchical clustering dividing the patient population into four groups each as shown in Fig. 4. Step 2: The clusters that differentiate the patients with favorable outcome from those with unfavorable outcome were identified by permutations of all 2:2 or 1:3 cluster combinations to identify the cluster combinations that resulted in the lowest *p*-value in log-rank analysis (raw data of this permutation analysis in Additional file 5: Table S4). Step 3: Finally, the ELIMA.score was defined as the sum of the separate LBA assignments. The calculation of the ELIMA.score in the samples is shown in Additional file 5: Table S4. The ELIMA.score can be understood as follows: a given patient had an ELIMA.score of 0 when they were not in the prognostically worst cluster for any of the LBAs; they had an ELIMA.score = 1 when they were in the prognostically worst cluster for one of the LBAs. Patients had an ELIMA.score = 2 when they were in the prognostically worst cluster for two of the LBAs, and so forth (further explanations are available in Additional file 2).

Singular value decomposition (SVD) was used to identify the singular vectors of the dataset, which in our case correspond to its most significant parameters [23]. To assess the dependence of one LBA on the other, we used mutual information [24]. In general, the higher the mutual information, the greater the ability of one LBA to describe the other (further explanations are available in Additional file 2).

Lloyd’s k-means clustering was undertaken for the sake of comparing its findings with those obtained by hierarchical clustering [25]. To obtain the appropriate number of clusters for a given dataset, we used the elbow method [26]. This method resulted in the curves shown in Additional file 6: Fig. S1.

Graph-theoretic analysis was conducted using the open source software Gephi [27]. This analysis was separately performed for all four clusters obtained using k-means clustering. The Yifan Hu (attraction–repulsion) algorithm was used to adjust the layout of the resulting network [28]. Then, a topology filter based on degree range was used to highlight the prominent nodes. Betweenness centrality, which is a measure of how often a node appears on the shortest paths between nodes in the network [29], was computed for each node to identify the parameters within a given cluster that play a salient role.

Detailed descriptions of all of the statistical approaches are available in Additional file 2. Diagrams were computed with R, using the packages *base* [30], *ggplot2* [31], *heatmap.plus* [32], *hmisc* [33], *infotheo* [34], *pca3d* [35], *shiny* [36], *stats* [30], *survival* [37], and *venndiagram* [38] (R version 3.6.1), Gephi (version 0.9.2), OriginPro version 2019 (OriginLab Corporation), and Microsoft Excel (Microsoft Corporation).

## Results

### Multi-modal liquid biopsy approach with four liquid biopsy analytes

ELIMA’s multi-parametric approach originally included the analysis of five LBAs (ELIMA means *five* in Hawaiian) from the same blood sample with minimized blood volume, namely EV mRNA, CTC mRNA, CTC gDNA, cfDNA from CTC-depleted blood (Fig. 1), and cfDNA from whole blood. However, since a direct comparison of cfDNA variants from whole blood and matched CTC-depleted blood revealed no significant differences in either qualitative or quantitative measures [20], cfDNA from whole blood was excluded from the final integration of the ELIMA project results.

The use of the same multi-marker qPCR for mRNA profiling of CTCs and matched EVs, and the use of the same targeted UMI-based panel for deep sequencing of cfDNA and matched CTC gDNA, guaranteed a meaningful comparison of the matched LBAs on a transcriptomic and genomic level.

Since the workflow utilizes material that would usually have been discarded (CTC-depleted blood and mRNA-depleted CTC lysate), isolation and analysis of all four LBAs has successfully been established from only 18 ml of blood.

### A large dataset

The patient cohort consisted of 26 MBC patients with HR+ HER2− status on the primary tumor tissue (Additional file 1: Table S1). Blood was drawn at the time of disease progression. At the time of data evaluation, 5/26 patients were alive with a median follow-up time of 113 months (interquartile range (IQR) 97).

The heatmap, plotting the clinical data of the cohort and the alterations in all 68 parameters divided into four different LBAs (Additional file 7: Fig. S2), depicts this large dataset. A few observations were directly obvious just by studying the heatmap. For example, the noticeably high prevalence of CTC gDNA variants, especially *MUC16* CTC gDNA variants. *mTOR* overexpression was the most common alteration in the CTC mRNA fraction, and *AURKA* overexpression signals showed the highest prevalence in the EV mRNA fraction. *ERBB2* and *ERBB3* variants or overexpression signals were tested in all four LBAs but were only present in the two CTC fractions (CTC gDNA and CTC mRNA; Additional file 7: Fig. S2).

### Sensitivity and number of signals per patient

**Fig. 2:**
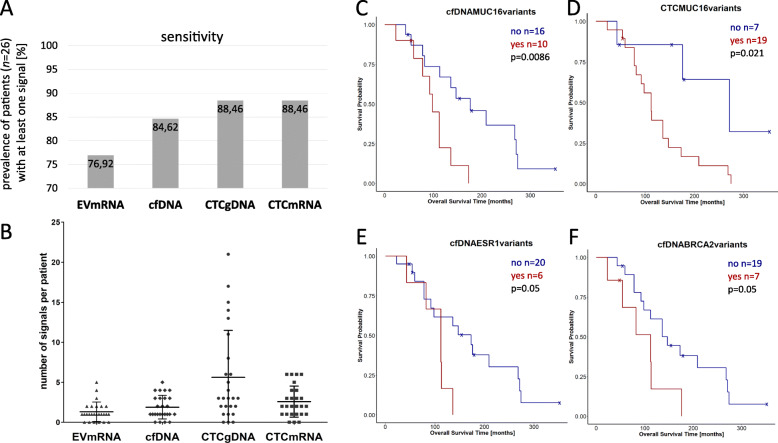
Sensitivity of the four single liquid biopsy analytes and Kaplan–Meier curves of the individual parameters. **a** Sensitivity defined by the prevalence of patients (in total *n*=26) with at least one variant (cfDNA or CTC gDNA) or one overexpression signal (CTC mRNA or EV mRNA). **b** The number of variants or overexpression signals per patient in each individual analyte was compared. The mean and standard deviation were indicated as solid lines and whiskers, respectively. **c**–**f** Data of patients with at least one variant/signal are depicted in red. Only significant correlations [calculated by log-rank (Mantel–Cox) test (*p*-value ≤ 0.05)] are shown. cfDNA variants in three genes (**c**, **e**, **f**) and *MUC16* variants in CTCs (**d**) showed a significant correlation with OS

Among the patients, 77% (20/26) showed at least one overexpression signal in EV mRNA, whereas 88% (23/26) had at least one overexpression signal in CTC mRNA or one CTC gDNA variant (Fig. 2a). Consequently, the sensitivity was higher in the two CTC fractions when compared to EV mRNA and cfDNA (85%; 22/26).

The range of the number of CTC gDNA variants per patient was high. The mean number of alterations per patient (± standard deviation) was the highest for CTC gDNA (5.62 ± 5.87), followed by CTC mRNA (2.58 ± 1.94), cfDNA (1.88 ± 1.48), and EV mRNA (1.31± 1.23) (Fig. 2b), respectively.

### Prognostic value of individual parameters

All parameters occurring in more than two patients were correlated with OS. Four parameters showed (at least a borderline) significance (*p* ≤ 0.05) with OS. Three out of these four parameters were cfDNA parameters and one was a CTC gDNA parameter (Fig. 2c–f). More specifically, prognostic value was documented for *MUC16* variants in cfDNA (*p*=0.0086) and CTC gDNA (*p*=0.021; Fig. 2c, d). *ESR1* (*p*=0.05) and *BRCA2* (*p*=0.05) variants in cfDNA showed borderline significance for a correlation with OS (Fig. 2e, f). In contrast, none of the CTC mRNA and EV mRNA parameters was significantly correlated with OS.

### Actionable signals for targeted therapy decisions

To evaluate the usefulness of the multi-parametric approach for targeted therapy decisions, a literature research was employed to define actionable signals whose presence might skew the treatment decision in favor of a targeted therapy (Additional file 8: Table S5 [39–45];). In each fraction, seven out of 17 parameters were defined as actionable, namely: *AKT1*, *BRCA1*, *BRCA2*, *PIK3CA*, *ESR1*, *ERBB2*, and *PTEN* variants, and *AR*, *AURKA*, *ERCC1*, *ERBB2*, *ERBB3*, *PIK3CA*, and *SRC* overexpression signals.

**Fig. 3:**
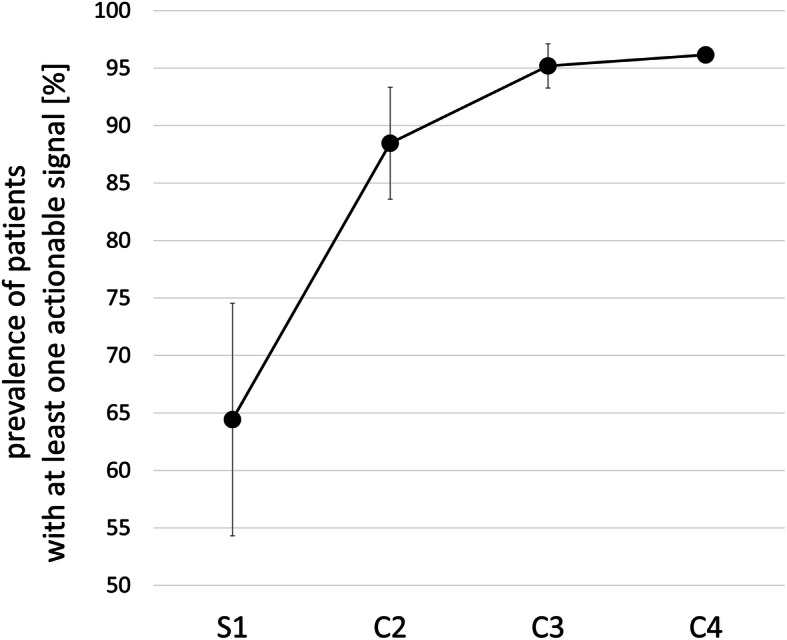
Prevalence of actionable signals. The prevalence of patients (among 26 patients) with at least one actionable signal is plotted against the evaluated analyte (combination). S1, each individual analyte; C2, any combination of two analytes; C3, any combination of three analytes; C4, a combination of all four analytes. Whiskers represent the standard deviation

Within each individual LBA, a similar fraction of patients [50% (EV mRNA) to 73% (CTC mRNA)] was identified with at least one actionable signal. The relevance of a multi-parametric strategy in therapy decision-making was validated by an increase in the percentage of patients with at least one actionable signal with an increase in the number of LBAs combined: a combination of two LBAs resulted in 81–92%, a combination of three LBAs resulted in 92–96%, and all four LBAs resulted in 96% of patients with actionable signals, respectively (Fig. 3).

### Hierarchical clustering and the ELIMA.score

**Fig. 4:**
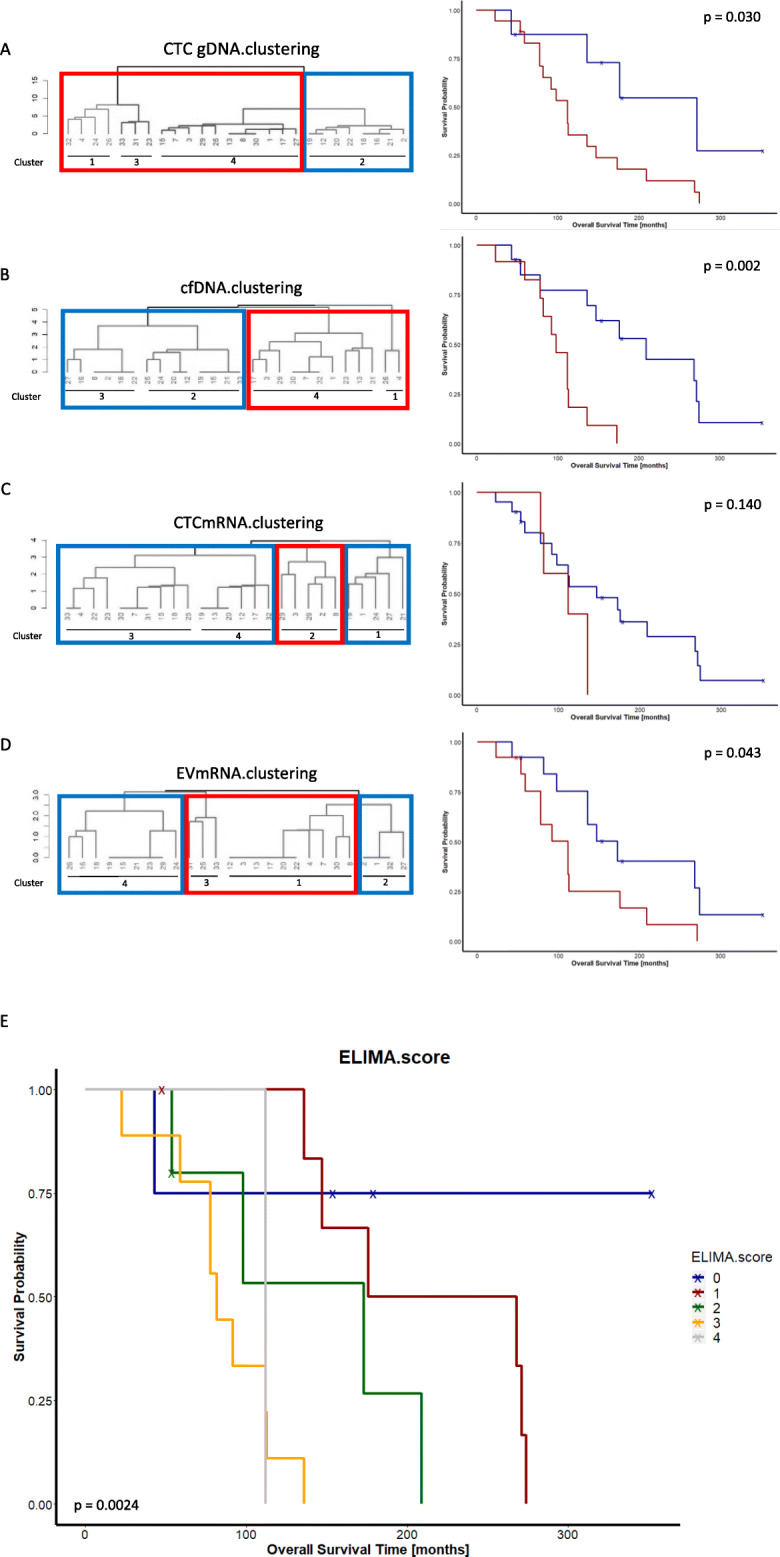
Hierarchical clustering and the prognostic value of the clusters. All LBAs were analyzed individually by means of hierarchical clustering by dividing the patient population into four groups. The clusters that differentiate the patients with favorable outcome (marked in red) from those with non-favorable outcome (marked in blue) were identified by permuting all cluster combinations to identify the cluster combination that resulted in the lowest *p*-value in log-rank analysis. Kaplan–Meier curves of the best permutation within each analyte are depicted and showed prognostic value (except for CTC mRNA-based clustering). A combination of the clustering results of all analytes, defined as the ELIMA.score, revealed good prognostic relevance (**e**)

Within each fraction, patients were clustered using hierarchical clustering by setting the number of clusters to four (Fig. 4a–d). To differentiate patients from one another based on the greatest difference in OS, permutations of the cluster combinations were conducted within each fraction in order to select the combination of patient clusters whose correlation with OS was the highest (Fig. 4a–d). Except for clustering based on CTC mRNA, patient clusters that were significantly correlated with OS were identified in all other fractions (Fig. 4a, b, d). A combination of these fraction-specific data—by integrating the clustering results of all fractions into a global ELIMA.score (see Additional file 2 for a detailed explanation)—resulted in a highly significant prognostic value (*p*=0.0024) (Fig. 4e). The OS decreased with an increase in the ELIMA.score and thereby underscores its prognostic value.

### Mutual information and the Euler diagram

The combination of clusters obtained by hierarchical clustering described in the section “Hierarchical clustering and the ELIMA.score” was carried out based on prior knowledge about the OS correlations within the LBAs, which—one could argue—carried with it a degree of subjectivity. In order to assuage such concerns, in what follows, the LBAs were evaluated using more objective methods to examine their interdependence.

**Fig. 5:**
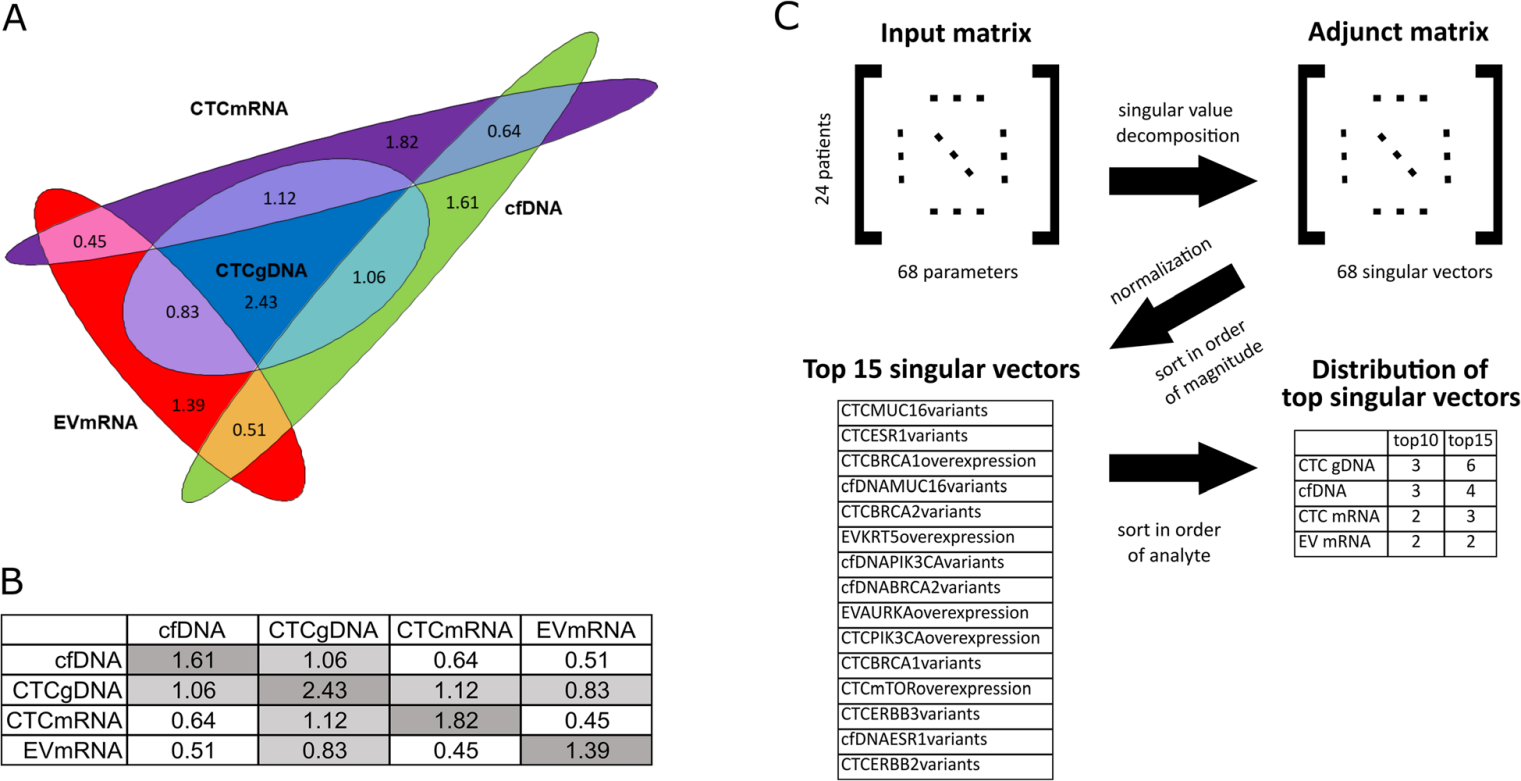
Mutual information (**b**) illustrated by an area-proportional Euler diagram (**a**) and singular value decomposition (**c**). **a**, **b** Pairwise comparison revealed the greatest overlap between information contained in CTC gDNA with the information contained in the other three analytes (threshold >0.8). The mutual information values were found to be rather low. Each analyte therefore added information that was absent in the other analytes. **c** The steps to identify the 15 strongest singular vectors representing parameters of all four analytes. Singular value decomposition of the input matrix resulted in an adjunct matrix of singular vectors. Normalization and sorting according to magnitude revealed that the 15 strongest singular vectors originated from all four analytes

With the intention of carrying out k-means clustering, the elbow method was employed to examine whether stable clusters could be formed using either individual analytes or when all the four analytes were combined and also to find the optimal number of clusters [26] (Additional file 2). Analysis of individual LBAs using the elbow method showed that only CTC gDNA-based clustering could result in the formation of stable clusters (Additional file 6: Fig. S1), which led to the question: “if stable clusters can be formed using only one of the LBAs (CTC gDNA), does it imply that the other LBAs are dependent on it?” To answer this question, we used mutual information to assess dependence between the LBAs [24] by a pairwise comparison of the individual LBAs (Fig. 5b). In general, the higher the mutual information, the greater the ability of one LBA to describe the other.

The extent of overlap between the individual LBAs based on the mutual information values is depicted in the Euler diagram (Fig. 5a). Since all the mutual information values were non-zero, we can conclude that none of the LBAs is independent of the others. However, from the relatively low mutual information values, we see that none of the LBAs can accurately describe the entire dataset on their own. This further underscores the additive nature of all four fractions. From the areas of overlap (Fig. 5a) and the highlighted cells in Fig. 5b, we construe that CTC gDNA is more dominant than the other LBAs in this dataset but since its dominance is not absolute, the other LBAs are necessary if one seeks to maximize the information that can be gathered from this data.

### The analytes and their strongest singular vectors

Then, the question “which of the 68 parameters (contained in the four LBAs) are the most influential” arose. This was addressed by using SVD to identify the singular vectors of the dataset [23]. The 10 most influential singular vectors were found distributed almost equally across all four fractions, while the 15 strongest singular vectors showed a relative dominance of the CTC gDNA fraction over the three other fractions because 40% of the top 15 singular vectors originated from CTC gDNA (Fig. 5c), thereby validating the findings in the section “Mutual information and the Euler diagram” that all LBAs should be considered if one seeks to maximize the information that can be gathered from this data.

### Results of k-means clustering based on a combination of all analytes

**Fig. 6:**
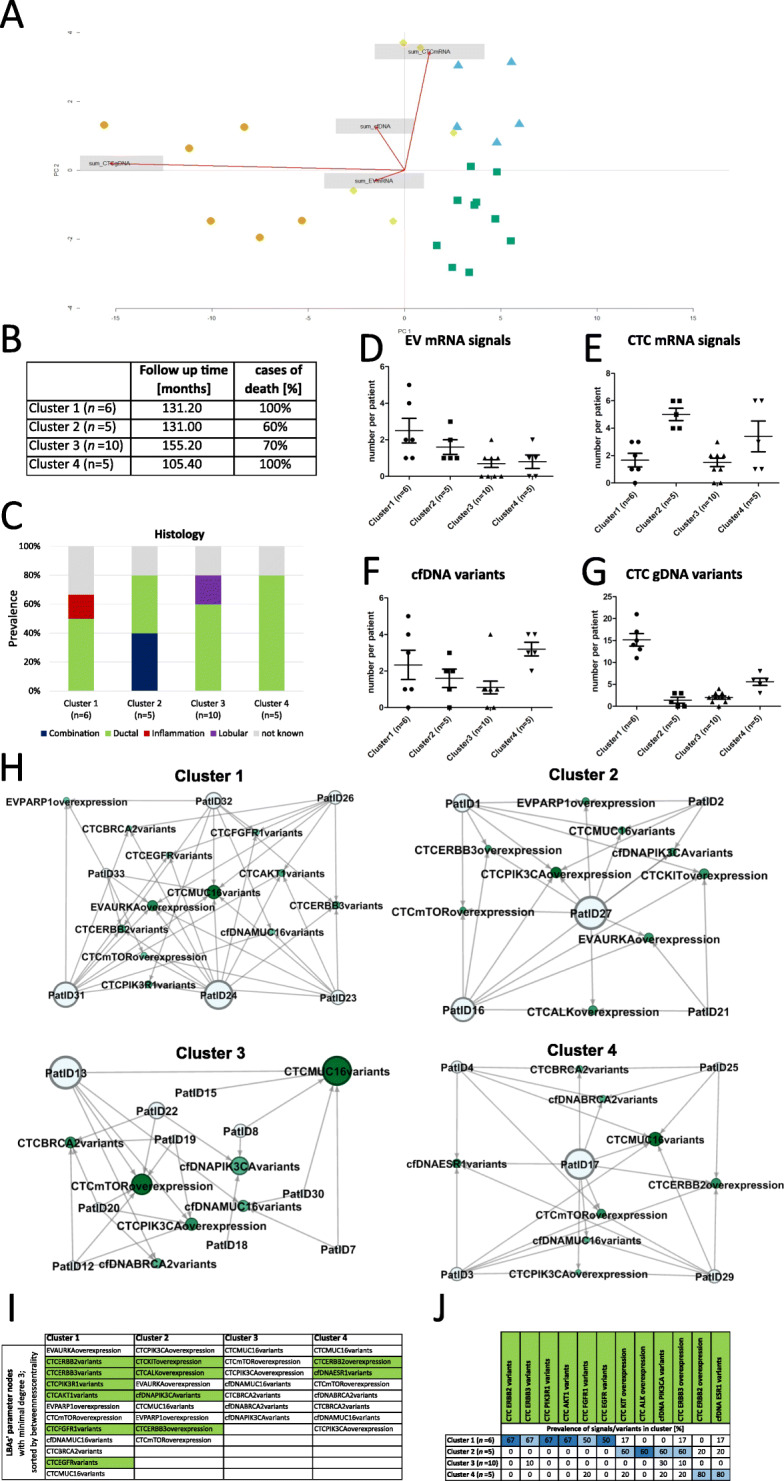
k-means clustering results based on the data from all four analytes and graph-theoretic analysis. **a** A 2D principal component analysis (PCA) plot illustrates the clusters. **b** The cluster size, follow-up time, and cases of death are listed. **c** Correlations of clusters with the tumor histology revealed that patients with a tumor histology other than the ductal type clustered together. **d**–**g** Number of alterations observed within the four analytes, grouped according to clusters formed based on all analytes. **h** Networks for each of the four clusters illustrate nodes with a degree ≥ 3 and directed edges between patients and parameters. The sizes of the nodes are proportional to the value of the (undirected) betweenness centrality and the intensity of the shade of green is proportional to the number of incoming edges. **i** Parameter nodes shown within the four networks were sorted based on their betweenness centrality. The 12 parameter nodes found in only one of the four networks were marked in light green. **j** The prevalence of signals/variants in the 12 unique parameter nodes (in **i**) was tabulated based on their occurrence in the four clusters. The cluster with the highest prevalence of a given parameter was marked in light blue and matched with the unique parameter nodes in **i**. Signals/variants of parameters found exclusively in just one cluster were marked in dark blue

As mentioned in the section “Mutual information and the Euler diagram”, we decided to employ k-means clustering to analyze the data without using prior information about OS correlations. In the sections “Mutual information and the Euler diagram” and “The analytes and their strongest singular vectors,” we examined the scenarios in which stable clusters could be formed and also examined the degree of dependence between the LBAs (Fig. 1). Here, we document the results of k-means clustering (Fig. 6).

In the sections “Mutual information and the Euler diagram” and “The analytes and their strongest singular vectors”, we found that a combination of all four analytes resulted in stable clusters using the elbow method (Additional file 6: Fig. S1) and that the results of the mutual information analysis as well as SVD indicated that clustering using all four analytes may be more informative than using the most suitable individual analyte, CTC gDNA. Consequently, we then carried out k-means clustering based on all four LBAs (Fig. 6a, b).

Interestingly, all patients with lobular histology subtype (*n*=2) and combinational histology subtype (*n*=2) clustered together in separate clusters (cluster 2 and cluster 3), while the patient with inflammatory BC clustered together with patients with ductal or unknown subtype in cluster 1 (Fig. 6c).

Subsequently, experimental liquid biopsy parameters within the resulting clusters were also compared (Fig. 6d–g). Cluster 1 was characterized by a large number of CTC gDNA variants, while the mean number of cfDNA variants was the highest in cluster 4. Though both cluster 2 and cluster 3 harbored a small number of CTC gDNA variants, cluster 2 contained a higher mean number of CTC mRNA signals when compared to cluster 3.

### Graph-theoretic analysis

To examine the salience of specific parameters within the clusters formed using k-means clustering, a graph-theoretic analysis was conducted (Fig. 6h). Importantly, only nodes with at least three edges were included to identify the most relevant nodes in the network. A comparison of the nodes, representing the parameters, within the four networks resulted in the identification of CTC *MUC16* variants and CTC *mTOR* overexpression as salient nodes in each network, but also resulted in the identification of parameter nodes unique to one of the four networks (Fig. 6i light green; 12 in total). We observe that the network of cluster 1 was predominantly characterized by the presence of CTC variants and is the only network that contained *ERBB2* variants in CTCs. The network of cluster 2 mainly consisted of CTC mRNA parameters and CTC *PIK3CA* variants, and the network of cluster 4 contained the *ESR1* variants in cfDNA and CTC *ERBB2* overexpression nodes. A comparison of these findings with the raw data set revealed that certain parameters, found to be unique nodes in a given cluster, remained exclusive in their signal occurrence within the given cluster (CTC variants of *ERBB2*, *PIK3R1*, *AKT1*, *FGFR*, and CTC *ALK* overexpression, Fig. 6j; dark blue). The other parameters, found to be unique nodes in a given cluster, were found to be at least highly abundant features (Fig. 6j; light blue) with a prevalence of ≥50%.

## Discussion

Liquid biopsy is currently gaining momentum as a valuable source for cancer detection, therapy monitoring, and treatment decision-making in oncology [12, 46–48]. However, most of these strategies utilized single LBAs. Here, we established a workflow to isolate and analyze four LBAs, namely CTC mRNA, CTC gDNA, EV mRNA, and cfDNA, from a minimized blood volume. With this successful protocol, in this hypothesis-generating pilot study, we can now answer questions about whether the information in one analyte can be conveyed by another analyte, or whether the information individual LBAs provide is unique, and ultimately, investigate whether it is worth conducting a multi-parametric liquid biopsy test in clinical practice.

Some studies have already compared the value of using cfDNA and CTCs for therapy management. The combination of cfDNA and CTC counts improved sensitivity and specificity as a diagnostic tool in non-MBC patients [5, 49]. A decrease in ctDNA and CTC levels from the baseline to the second cycle of paclitaxel and bevacizumab in HER2− MBC patients was independent prognostic markers, but with a stronger value for ctDNA when compared to CTCs [50]. The comprehensive mutational analysis of cfDNA and CTCs revealed the additive value of the analytes [14, 51]. Variant analysis in CTCs was shown to be able to identify newly emerging resistance mutations in contrast to cfDNA, where resistance mutations might only be detected after apoptosis of the cells harboring new alterations [51], thereby highlighting the potential benefits of variant analysis in CTCs over cfDNA. The case study of a HR+ MBC patient with serial liquid biopsies across treatment over 4 years showed the correlation of single CTC and cfDNA copy number variants and mutations, but the authors argued that only the analysis of variants in single CTCs deconvolutes the subclonal evolution in cellular resolution [52].

The technical issues of a multi-parametric liquid biopsy approach, including CTCs, cfDNA, EVs, and miRNA, were recently studied by Schneegans et al. as part of the Cancer-ID consortium [53]. They showed the importance of the pre-analytical variables, especially the choice of a blood collection tube for reliable data analysis. In cooperation with several companies specialized in the evaluation of individual LBAs, Hodara et al. determined alterations in multiple liquid biopsy reservoirs of prostate cancer patients from 22.5 ml blood [54]. Hodara et al. reported a 13.8% overlap between variants of CTCs and cfDNA, while we reported a 28% overlap for these two LBAs in MBC patients [14]. It is worth emphasizing that the ELIMA project examined not just tumor-specific variants but also variants in germline controls, e.g., 48% of cfDNA and 35% of the CTCgDNA variants were detected in the germline control (Additional file 4: Table S3). Another recent multi-analyte liquid biopsy study using 12.5 ml blood from 19 prostate cancer patients showed an increase in the probability of obtaining tumor-related information [55]. Combinational analysis of transcripts in CTCs, the same transcripts in whole blood, and focal amplifications in cfDNA led to the identification of resistance mechanisms against prior therapy in a larger fraction of patients when compared to the evaluation using a single analyte [55]. The reported sensitivity of only one analyte was around 50% and the sensitivity of the multi-analyte strategy was 89% and is similar to the results in this paper from 26 MBC patients and four LBAs. Yang et al. recently studied the feasibility of a multi-modal liquid biopsy approach, which included tumor-associated EV miRNA and mRNA, cfDNA, cfDNA *KRAS* mutations, and CA19-9, for early diagnosis of pancreatic cancer in 204 subjects [56]. They developed an initial classification model using 14 biomarker candidates, trained a machine-learning model, and achieved a sensitivity of 88% and a specificity of 95% in the validation cohort, thus validating the benefits of a multi-modal approach using a completely different method of analysis. A multi-modal liquid biopsy approach has also been tested as a predictive panel supporting therapy decisions for immune therapy in lung cancer patients. The integrated multi-sourced information from ctDNA and circulating immune cells (called the DIREct-On approach therein) resulted in better differentiation of patients with durable response from those without durable response (before initiation of therapy) when compared to using information solely from individual LBAs [57].

In the context of the ELIMA project, we have already demonstrated the additive value of using CTC mRNA profiling in addition to matched EV mRNA profiling utilizing blood from HR+ HER2− MBC patients [13] and the small overlap between identical variants in cfDNA and matched CTC gDNA [14]. This work therefore bridges the gap between transcriptomic and genomic information, albeit in a small cohort (*n*=26) and without a validation cohort.

The high prevalence of *MUC16* variants in CTCs is striking (Additional file 7: Fig. S2), and so is the correlation of *MUC16* variants (in both CTCs and in cfDNA) with decreased OS (Fig. 2c–f). *MUC16* variants have frequently been reported in most cancer types [58, 59] but considering the long coding sequence of this gene, the mutational heterogeneity of *MUC16* is not elevated in BC [59, 60]. The other individual parameters which have a significant correlation with OS, namely *ESR1* and *BRCA2*, are genes that are frequently discussed and harbor great clinical relevance in BC [39].

That is the reason why *AKT1*, *BRCA1*, *BRCA2*, *PIK3CA*, *ESR1*, *ERBB2*, and *PTEN* variants were described as harboring at least Tier III A level of evidence for targeted therapy approaches by ESMO [39] and were selected as actionable signals, despite generalizing an effect of mutations in one gene independent of their position. We defined *AR*, *AURKA*, *ERCC1*, *ERBB2*, *ERBB3*, *PIK3CA*, and *SRC* overexpression signals as actionable signals by literature research as well; this definition, however, is highly speculative, because most of the references showed the predictive value only in animal models [40–44]. Analysis of individual LBAs resulted in a discovery of actionable signals in approximately half of all patients, while a combination of two, three, or all four LBAs revealed a dramatic increase in the prevalence of patients with at least one actionable marker, hence providing evidence for the clinical relevance of this multi-parametric approach.

The ELIMA.score provides additional insights into the value of the multi-parametric approach. Grouping patients according to their liquid biopsy data within a given LBA (CTC gDNA, cfDNA, or EV mRNA) resulted in a division of patients into cohorts with shorter versus longer OS, but combining these hierarchical clustering results underscored the high prognostic value of integrating them.

In initial observations, CTC gDNA showed the highest sensitivity and the highest number of signals per patient. It was also the only LBA suitable for obtaining stable clusters using k-means clustering (according to the elbow curves) and was the only LBA that—based on the mutual information values—contained the greatest amount of information about the other LBAs. However, based on the fact that the absolute values of mutual information were rather low (< 3), it was found that using CTC gDNA alone seems not to be sufficient.

The strongest singular vectors define the most influential parameters within this large dataset, and interestingly, among the 10 strongest singular vectors, parameters from all four LBAs were equally distributed, implying that at least some factors might have clinical relevance despite the analyte—as a whole—having low sensitivity. The 15 strongest singular vectors point to a relative dominance of CTC gDNA among the LBAs since 40% of the 15 strongest singular vectors originated from CTC gDNA. However, since a majority of the strongest singular vectors stem from the other three LBAs, this analysis also found that the information obtained from CTC gDNA could not describe the overall picture alone.

The surplus information, obtained from the multi-parametric liquid biopsy approach by integrating the four LBAs, was mirrored not only by the prognostic value of the ELIMA.score and the improved sensitivity for actionable markers, but also by the low mutual information values and a fairly even spread of data points across the dimensions when k-means clustering based on all four LBAs was carried out. k-means clustering and graph-theoretical analysis of the resulting clusters further highlighted the differences and identified some unique features across the different LBAs in the four clusters, thereby accentuating the relevance of the integration of all four LBAs.

## Conclusions

High sensitivity, a high mean number of CTC gDNA variants, and the elbow curves used for k-means clustering revealed the CTC gDNA fraction to be comparatively more influential than the other three LBAs tested.

However, the ELIMA.score, mutual information, the prevalence of the strongest singular vectors in all the LBAs, k-means clustering based on all four LBAs, and the prevalence of actionable signals in all four LBAs uncovered their additive prognostic value and showed that—though influential—using CTC gDNA alone does not suffice.

We conclude that CTC gDNA, CTC mRNA, EV mRNA, and cfDNA are complementary rather than competitive and a multi-parametric liquid biopsy approach like the ELIMA project, which simultaneously interrogates diverse LBAs, is worth using in clinical practice, because it enables the generation of a high-resolution snapshot of the genomic and transcriptomic disease complexity.

## Supplementary Information

**Additional file 1: Table S1**. Patient characteristics.

**Additional file 2: Supplementary methods**.

**Additional file 3: Table S2**. Sequencing quality parameters.

**Additional file 4: Table S3**. Called cfDNA and CTCgDNA variants, their corresponding allele frequencies and indication of identical variants detected in the germline control.

**Additional file 5: Table S4**. Hierarchical clustering results, their permutations, and the ELIMA.score.

**Additional file 6: Fig. S1**. Elbow curves illustrating the ability of individual analytes and the combination of all four analytes to form stable clusters.

**Additional file 7: Fig. S2**. Heatmap.

**Additional file 8: Table S5**. Literature research for actionable marker in breast cancer.

## Data Availability

Original raw sequencing data are available at the European Nucleotide Archive with the study accession number PRJEB39331 (https://www.ebi.ac.uk/ena/browser/view/PRJEB39331) [22]. The sequencing quality parameters for each sample are summarized in Additional file 3: Table S2. All called variants and their corresponding allele frequencies are listed per patient and per analyte in Additional file 4: Table S3. The binary data (variant/overexpression signal yes/no) of all patients (*n*=26) within all LBAs are listed in Additional file 7: Fig. S2.

## Abbreviations

BC: Breast cancer
cfDNA: Cell-free DNA
ctDNA: Cell-free tumor DNA
CTCs: Circulating tumor cells
ELIMA: Evaluation of multiple Liquid biopsy analytes In Metastatic breast cancer patients All from one blood sample
ER: Estrogen receptor
EVs: Extracellular vesicles
gDNA: Genomic DNA
HR+: Hormone receptor-positive
IVA: Ingenuity Variant Analysis
IQR: Interquartile range
LBAs: Liquid biopsy analytes
mRNA: Messenger RNA
MBC: Metastatic breast cancer
OS: Overall survival
PR: Progesterone receptor
PCA: Principal component analysis
RT-qPCR: Real-time quantitative PCR
RECIST: Response Evaluation Criteria in Solid Tumors
SVD: Singular value decomposition
UMIs: Unique molecular indices

## List of genes

AKT1: AKT serine/threonine kinase 1
AKT2: AKT serine/threonine kinase 2
AR: Androgen receptor
AURKA: Aurora kinase A
BRCA1: BRCA1 DNA repair associated
BRCA2: BRCA2 DNA repair associated
EGFR: Epidermal growth factor receptor
EpCAM: Epithelial cell adhesion molecule
ERCC1: ERCC excision repair 1, endonuclease non-catalytic subunit
ERCC4: ERCC excision repair 4, endonuclease non-catalytic subunit
ERBB2: erb-b2 receptor tyrosine kinase 2 coding for the HER2 protein
ERBB3: erb-b2 receptor tyrosine kinase 3
ESR1: Estrogen receptor 1
FGFR1: Fibroblast growth factor receptor 1
GAPDH: Glyceraldehyde-3-phosphate dehydrogenase
KIT: KIT proto-oncogene receptor tyrosine kinase
KRT5: Keratin 5
KRAS: KRAS proto-oncogene, GTPase
MET: MET proto-oncogene, receptor tyrosine kinase
mTOR: Mechanistic target of rapamycin
MUC16: Mucin 16, cell-surface associated
NOTCH1: Notch 1
PARP1: Poly(ADP-ribose) polymerase 1
PIK3CA: Phosphatidylinositol-4,5-bisphosphate 3-kinase catalytic subunit alpha
PIK3R1: Phosphoinositide-3-kinase regulatory subunit 1
PTEN: Phosphatase and tensin homolog
PTGFR: Prostaglandin F receptor
PTPRC: Protein tyrosine phosphatase, receptor type, C (also known as CD45)
SOX17: SRY-box transcription factor 17
SRC: SRC proto-oncogene, non-receptor tyrosine kinase
TGFB1: Transforming growth factor beta 1
