# Supplementary material for: Integrative statistical analyses of multiple liquid biopsy analytes in metastatic breast cancer
Source: Genome Med. 2021 May 17;13:85. doi: 10.1186/s13073-021-00902-1 (PMC8130163; doi:10.1186/s13073-021-00902-1)
Supplement: Supplementary file 2 — Additional file 2: Supplementary methods. [file 13073_2021_902_MOESM2_ESM.docx]

**Additional file 2: Supplementary methods**

Patient population characteristics and eligibility criteria

The study was conducted at the Department of Gynecology and Obstetrics, in collaboration with the Department of Medical Oncology, both at the University Hospital Essen, Germany, with the Marienhospital Bottrop, Germany (for specimen recruitment) and with QIAGEN GmbH, Hilden, Germany (for library preparation and sequencing analysis). In accordance with the Declaration of Helsinki, written informed consent was obtained from all participants at enrollment and specimens were collected using protocols approved by the Ethics Committee of the University Hospital of Essen (12-5265-BO). In total, blood samples from 26 MBC patients were studied between August 2015 and May 2019 of which some patient samples have already been used for molecular characterization of matched LBAs [13,14]. All participants were ≥18 years, had Eastern Cooperative Oncology Group (ECOG) scores for performance status of 0-2, no severe, uncontrolled co‑morbidities, or medical conditions, and no second malignancies. Prior treatment, radiation, all kinds of surgical intervention, or any other treatment of BC was permitted. MBC patients had estrogen (ER) and/or progesterone (PR) receptor-positive primary tumors [summarized as hormone receptor-positive (HR+)]. Furthermore, all included patients had primary tumors with a) <10 % of HER2 expressing tumor cells (DAKO score 0), or b) with HER2 expressing cells without complete membrane staining (DAKO score 1), or c) tumors with DAKO score 2, but without *ERBB2* overamplification (by in-situ hybridization) (n=22). Patients with ER-positive and/or PR-positive and HER2-negative metastases were also included if their ER, PR and HER2 status of the primary tumor was unknown (n=4). All patients showed a progressive MBC at the time of blood draw evaluated by visual staging. Patient characteristics are listed in Additional File 1: Table S1.

Sampling of blood, processing of plasma, and enrichment of circulating tumor cells

2 x 9 ml EDTA blood was collected in S-Monovettes^®^ (Sarstedt AG & Co), stored at 4°C, and processed within 4 hours. CTCs were isolated in duplicate from 5 ml of whole blood by positive immunomagnetic selection targeting EpCAM, EGFR, and HER2 (AdnaTest EMT-2/StemCell Select^TM^, QIAGEN) [13]. The CTC-depleted blood remaining after positive immunomagnetic selection [20] as well as the remaining blood not used for CTC isolation were centrifuged at 1841 x g for 8 min and plasma was frozen at - 80°C.

Isolation of extracellular vesicles and vesicular RNA

Thawed plasma was centrifuged at 3000xg for 8 min at room temperature and subsequently passed through a 0.8-µm-pore size syringe filter (Sartorius) to ensure removal of apoptotic bodies and cell debris. EVs were isolated from four ml of prefiltered plasma by affinity-based binding to a spin column [13,21]. The vesicles were bound onto a membrane independent of their size or cellular origin, and lysed on the column; subsequently, the total RNA was isolated and purified (exoRNeasy Kit, QIAGEN).

mRNA isolation and reverse transcription

The mRNA was isolated from the entire CTC lysates and from the total vesicular RNA eluates by Oligo(dT)_25_ beads (AdnaTest EMT-2/StemCell Detect^TM^, QIAGEN) [13]. The supernatant remaining from the CTC lysates after incubation with the Oligo(dT)_25_ beads, called the mRNA-depleted CTC lysate, was stored at -80°C. Purified mRNA was reverse transcribed (Adna Test EMT-2/StemCell Detect^TM^, QIAGEN) [12].

Quantitative PCR

The AdnaTest TNBC Panel prototype (QIAGEN), consisting of multi-marker RT-qPCR assays required transcript-specific pre-amplification and has already been described in detail [12]. Pre-amplified cDNA was analyzed in duplicates for one of the 17 transcripts (namely, *AKT2, AR, AURKA, BRCA1, EGFR, ERCC1, ERBB2, ERBB3, KIT, KRT5, MET, MTOR, NOTCH1, PARP1, PIK3CA, SRC, GAPDH*). RT-qPCR was performed with the StepOnePlus™ (Life Technologies) real-time system. Fluorescence readout was performed at 75°C. Additionally, melting curves were obtained. Potential PCR inhibition and contamination were checked, and data evaluation was performed according to previously published protocols [12]. In brief, transcripts not exclusively expressed in CTCs were normalized to the leukocyte-specific transcript *CD45* (also known as *PTPRC*). CTC and EV expression data of the patients were normalized to matched expression data of healthy donor controls and signals were analyzed binarily (overexpression yes/no). All MIQE criteria were met and have already been published in the Supplementary Material of [13].

Isolation and quantification of cfDNA

cfDNA was isolated using ≥1 ml plasma from CTC-depleted blood. The plasma sample volume used ranged from 1.2 ml – 6.2 ml (mean 4.2 ml; maximized plasma volume available). cfDNA isolation was conducted by affinity-based binding to magnetic beads (QIAamp MinElute ccfDNA Kit, QIAGEN), as previously described [20]. cfDNA yield was calculated for all fragments with lengths between 100 and 700 bp (assessed using the Agilent Chip High Sensitivity DNA).

Isolation of gDNA from CTCs

The mRNA-depleted CTC lysates were pooled in cases where two lysates were available from the CTC isolation in duplicate (n=23). The entirety of the mRNA-depleted CTC lysates was used to isolate the gDNA by the AllPrep DNA/RNA Nano Kit prototype (QIAGEN) as previously described [14].

Library construction

cfDNA and CTC gDNA were not amplified prior to library preparation. The libraries were constructed with a customized QIAseq Targeted DNA Panel Kit (QIAGEN) targeting all exonic regions of 17 genes (namely *AKT1, AR, BRCA1, BRCA2, EGFR, ERBB2, ERBB3, ERCC4, ESR1, KRAS, FGFR1, MUC16, PIK3CA, PIK3R1, PTEN, PTGFR* and *TGFB1)*. Library preparation using cfDNA or CTC gDNA was previously described in detail [14]. The preferred input amount for library preparation was in the range of 30-60 ng but cfDNA samples with a lower input were also included in the library preparation, and the entire CTC gDNA eluate (20µl) was used for library preparation with no prior quantification. Available germline controls (2 buffy coat DNA samples and 16 normal tissue DNA samples of 18/26 patients) were prepared for sequencing with the same customized QIAseq Targeted DNA Panel Kit (QIAGEN).

Sequencing

Libraries were quantified by qPCR and the quality was checked using Agilent Chip High Sensitivity DNA (Santa Clara) [14,22]. Libraries were diluted and CTC gDNA libraries were first analyzed by paired-end sequencing on an Illumina MiSeq instrument using the MiSeq Reagent Nano Kit v2 for up to 1 million paired-end reads to check the desired dilution and equal distribution of all libraries within the pool. All pooled libraries were analyzed by paired-end sequencing on an Illumina NextSeq instrument using the NextSeq 500/550 High Output Kit v2.5 with 2x150 bp reads using a custom sequencing primer (QIAseq A Read1 Primer).

Data analysis/ Bioinformatical analysis

Bioinformatical analysis of the raw sequencing data of cfDNA, CTC gDNA and the germline controls was performed on the basis of the pipeline previously described [14]. Sufficient sequencing quality of all samples was guaranteed by the exclusion of cfDNA libraries with fewer than 4 million read fragments, a unique molecular index (UMI) coverage lower than 400, and if less than 94% of the target region was covered with at least 5% of the mean UMI coverage. The latter criterion was also employed to exclude germline control libraries with poor sequencing quality. Input amount, library yield, and sequencing quality parameters for each sample are summarized in Supplementary Table 3. For analysis, we used the NGS Analysis service for QIAseq Targeted DNA Panels available at QIAGEN’s GeneGlobe, which allowed reliable variant calling based on UMI information. Ingenuity Variant Analysis (IVA; QIAGEN) was further used for annotation, scoring, filtering, and interpretation of the resulting variant files. All filter settings were described in detail [14]. The variant nomenclature used in this publication refers to the protein variant with the longest amino acid sequence.

Original raw sequencing data are available at the European Nucleotide Archive with the study accession number PRJEB39331 [23] and sequencing quality parameters are listed in Supplementary Table 3, whereas all called variants and their corresponding allele frequencies are listed per patient and per analyte in Supplementary Table 4.

Survival analysis

Kaplan-Meier estimator and Cox regression models were used to assess survival after first diagnosis (indicated as OS). Survival curves were compared using the log-rank (Mantel-Cox) test. Cox proportional-hazards regression analysis was used to estimate univariate and multivariate hazard ratios (wherever applicable) using the R packages *shiny*, *ggplot2*, and *survival* (R version 3.6.1).

Hierarchical clustering

Hierarchical clustering according to Ward’s method with Euclidean distance was conducted using the R packages *heatmap.plus* and *hmisc* (R version 3.6.1) as follows.

Step1: First, all LBAs were analyzed separately by means of hierarchical clustering dividing the patient population into four groups each as shown in Fig. 4.

Step2: The clusters that differentiate the patients with favorable outcome (all subsequently assigned the number 0) from those with unfavorable outcome (all subsequently assigned the number 1) were identified by permutations of all 2:2 or 1:3 cluster combinations to identify the cluster combinations that resulted in the lowest p-value in log-rank analysis. Since we represent the presence or absence of patients in the four clusters as a binary variables (favorable = 0, unfavorable = 1), we have 16 permutations (0 0 0 0 to 1 1 1 1 ) in total and they form the Galois field GF(16). The Galois field GF(16) is irreducible over GF(2), i.e., it is irreducible modulo 2. The presence of every additional cluster on GF(2) can be interpreted as the logical XOR operation (i.e., addition without carry). This implies that permutations like (1 0 0 0) and (0 1 1 1) are equivalent and – as a result – have the same p-value. Furthermore, since the equivalent permutations (0 0 0 0) and (1 1 1 1) do not help us differentiate between the clusters identified, they can also be excluded. This leaves us with the seven permutations which are sufficient to describe all variations possible (raw data of this permutation analysis in Additional File 5: Table S4).

Step3: Finally, the ELIMA Score was defined as the sum of the separate LBA assignments. The calculation of the ELIMA.score in the samples is shown in Additional File 5: Table S4. The ELIMA.score can be understood as follows: a given patient had an ELIMA.score of 0 when they were not in the prognostically worst cluster for any of the LBAs; they had an ELIMA.score = 1 when they were in the prognostically worst cluster for *one* of the LBAs. Patients had an ELIMA.score = 2 when they were in the prognostically worst cluster for *two* of the LBAs, and so forth.

Singular value decomposition

Singular value decomposition (SVD) was used to identify the singular vectors of the dataset [29]. Broadly speaking, SVD is similar to principal component analysis (PCA). The crucial difference between the two is that SVD can be applied to any matrix whereas PCA is applicable only to diagonalizable matrices. Since our input matrix was not diagonalizable, we used SVD. Practically, SVD was carried out using the R package *base* for the 24x68 input matrix (Fig. 5 C). The singular vectors were subsequently normalized by their respective magnitudes and sorted in descending order of their magnitude.

Mutual information calculation

Since the exact relationship between the LBAs is unknown, and we did not want to assume linearity and use measures of correlation, we used mutual information [28] to assess the dependence between the LBAs. In general, the higher the mutual information, the greater is the dependence of one LBA on the other and – consequently – the greater the ability of one LBA to describe the other. A mutual information of 0 indicates that the two LBAs being compared are independent of one another. Mutual information can take values in the interval $[0,\infty)$ and for two jointly discrete random variables $X$ and $Y$, it can be computed as:

$I\left( X:Y \right)= \sum_{y\in\mathcal{Y}} \sum_{x\in\mathcal{X}} p_{\left( X,Y \right)}\left( x,y \right)\log\left( \frac{p_{\left( X,Y \right)}\left( x,y \right)}{p_{\left( X \right)}\left( x \right)p_{\left( Y \right)}\left( y \right)} \right)$,

where $p_{(X,Y)}$ is the joint probability mass function of $X$ and $Y$, and $p_{(X)}$ and $p_{(Y)}$ are the marginal probability mass functions of $X$ and $Y$, respectively. This aforementioned calculation was carried out using the R package *infotheo*.

Lloyd’s k-means clustering

Lloyd’s k-means clustering is a commonly used clustering method which aims to partition ‘*n*’ observations into ‘*k*’ clusters while minimizing the within-cluster variances [26]. Since k-means clustering algorithms require the number of clusters (to be formed) as input, to obtain this information, we used the elbow method [27] to select the appropriate number of clusters for a given dataset (Fig. 1). For a given dataset, the elbow method expresses the percentage of variance as a function of the number of clusters and the ‘optimal’ number of clusters is the number beyond which the addition of a cluster does not lead to significant increase in the ability to explain the variance in the dataset. This method resulted in the curves shown in Supplementary Fig. 2. If an optimal number of clusters exists, the curve roughly resembles a bent arm and the location of the ‘elbow’ in this arm analogy gives the ‘optimal’ number of clusters. More formally, the ‘elbow’ ideally represents a stark point of transition where the value of the gradient between two consecutive points on one side of the point on the curve is significantly larger than the value of the gradient on the other side. The elbow method and the subsequent k-means clustering were carried out using the R package *stats*.

Graph-theoretic Analysis

Graph-theoretic Analysis was conducted using the open source software Gephi (version 0.9.2) [28]. This analysis was performed for each of the four clusters obtained using k-means clustering. The nodes consist of patients and parameters belonging to a given cluster. A directed edge connects a ‘patient’ node to a ‘parameter’ node if a given parameter is present in a given patient. To examine the network so formed, the Yifan Hu (attraction - repulsion) algorithm was used to adjust the layout of the network [29]. Then, a topology filter based on degree range was used in order to eliminate nodes with fewer than three edges (i.e., to filter out parameters occurring in fewer than three patients). Network statistics like the betweenness centrality were then computed for the undirected version of the graph [30]. The sizes of the nodes in the graph were then altered to reflect their betweenness centrality, which is a measure of how often a node appears on the shortest paths between nodes in the network. Subsequently, the nodes were colored based on in-degree, i.e., the larger the number of incoming edges, the deeper the shade of green. This enables us to identify the parameters within a given cluster which play a salient role.

Diagrams were computed with R, including the package *pca3d* and *venndiagram* (R version 3.6.1), Gephi (version 0.9.2), OriginPro version 2019 (OriginLab Corporation), and Microsoft Excel (Microsoft Corporation).
