# Supplementary material for: Integrative statistical analyses of multiple liquid biopsy analytes in metastatic breast cancer
Source: Genome Med. 2021 May 17;13:85. doi: 10.1186/s13073-021-00902-1 (PMC8130163; doi:10.1186/s13073-021-00902-1)
Supplement: Supplementary file 6 — Additional file 6: Fig. S1. Elbow curves illustrating the ability of individual analytes and the combination of all four analytes to form stable clusters. [file 13073_2021_902_MOESM6_ESM.pdf]

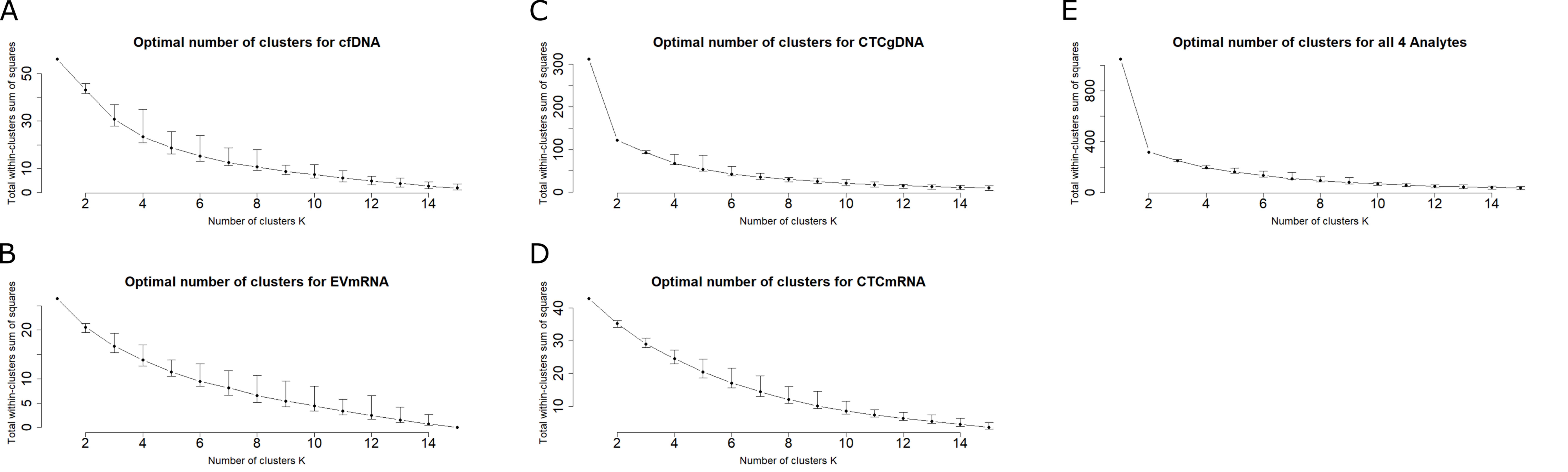

Additional file 6: Fig. S1: Elbow curves illustrating the ability of individual analytes and the combination of all four analytes to form stable clusters. CTC gDNA was the only analyte for which k-Means clustering resulted in stable clusters (optimal number of clusters = 6). The combination of all four analytes also resulted in stable clusters using k-Means clustering (optimal number of clusters = 4).
