# Supplementary material for: Integrative statistical analyses of multiple liquid biopsy analytes in metastatic breast cancer
Source: Genome Med. 2021 May 17;13:85. doi: 10.1186/s13073-021-00902-1 (PMC8130163; doi:10.1186/s13073-021-00902-1)
Supplement: Supplementary file 7 — Additional file 7: Fig. S2. Heatmap. [file 13073_2021_902_MOESM7_ESM.pdf]

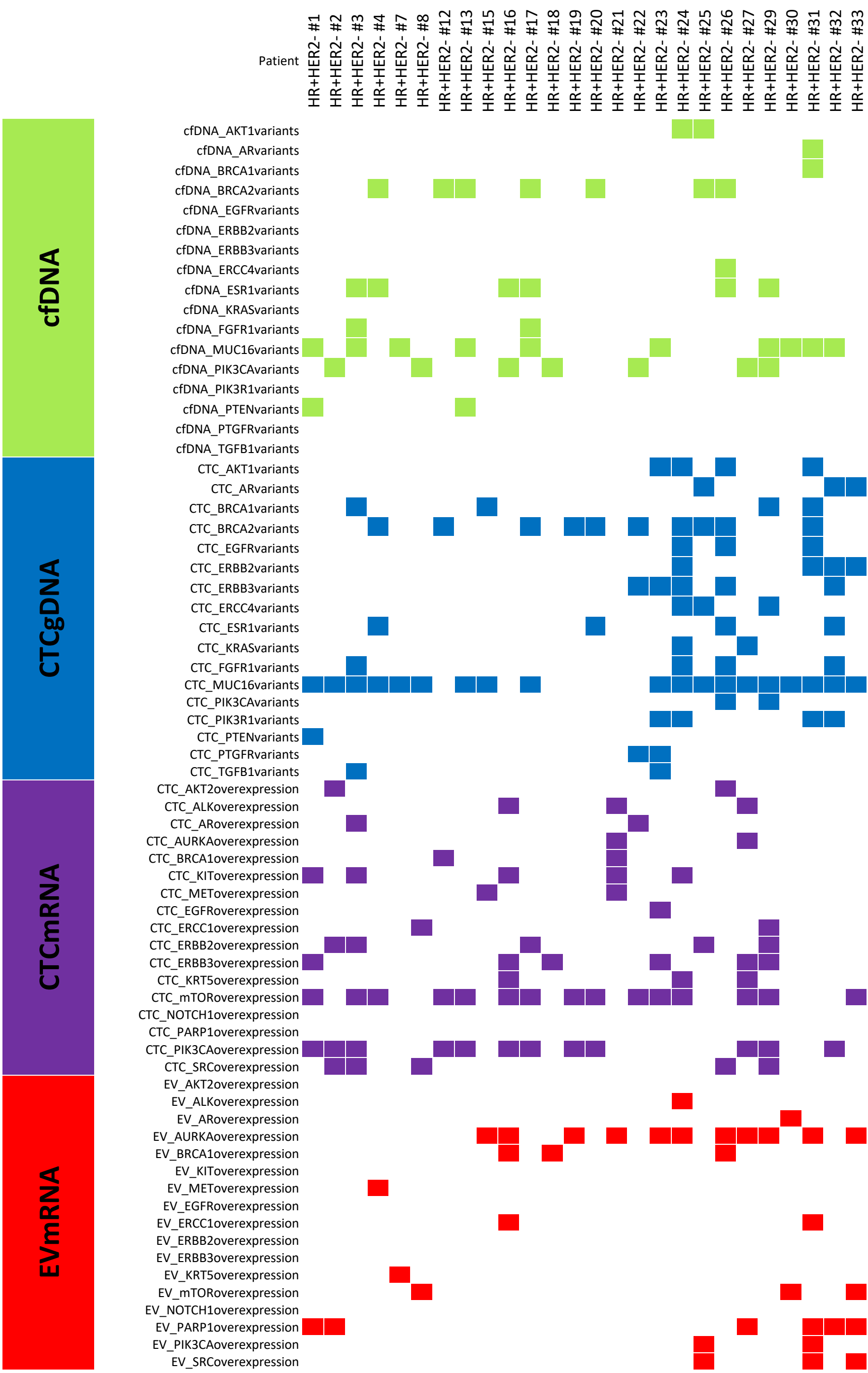

Additional file 7: Fig. S2: Heatmap. Experimental liquid biopsy data of all 26 HR+ HER2- MBC patients are depicted. Filled cells show the existence of at least one variant/overexpression signal. *mTOR* signals were the most prominent signals in the CTC mRNA fraction, *AURKA* signals were most prevalent in the EV fraction, and *MUC16* variants were common in cfDNA.
